# PROTEOSTASIS: NOVEL INSIGHTS AND TECHNOLOGIES

**DOI:** 10.1093/geroni/igac059.1665

**Published:** 2022-12-20

**Authors:** Constanza Cortes

**Affiliations:** University of Alabama at Birmingham, Birmingham, Alabama, United States

## Abstract

Skeletal muscle has recently arisen as a novel regulators of Central Nervous System (CNS) function and aging, secreting bioactive molecules known as myokines with proteostasis and metabolism-modifying functions in targeted tissues, including the CNS. Myokine secretion is heavily modified by exercise, suggesting that myokine signaling in the periphery may underlie the well document geroprotective benefits of exercise on the brain. The following studies address muscle proteostasis, a pathway highly activated during exercise, as a potential new regulator of the neurocognitive benefits of exercise. We have recently generated a novel transgenic mouse with enhanced muscle proteostasis via moderate overexpression of Transcription Factor E-B (TFEB), a powerful master regulator of cellular clearance and proteostasis. We have discovered that the resulting enhanced skeletal muscle proteostasis function can significantly ameliorate proteotoxicity and reduce neuroinflammation in the aging CNS. We derived cTFEB;HSA-Cre transgenic mice in the P301S MAPT background and we detected a significant reduction in hyperphosphorylated tau[AT8 phospho-tau antibody] in whole hippocampal lysates and in the dentate gyrus of cTFEB;HSACre;P301S mice compared to their single transgenic P301S littermate controls. Nanostring nCounter®AD panel analysis reveals displayed reductions in microglia activation modules in P301S MAPT/cTFEB:HSACre hippocampi, suggesting reduced neuroinflammation. We also determined that these CNS benefit sin P301S MAPT/cTFEB:HSACre mice were accompanied by activation of exercise-associated neurotrophic signaling and reduced markers of advancing tau-associated pathologies in the hippocampus. These provocative results suggest that enhanced skeletal muscle proteostasis modifies the accumulation of pathogenic tau isoforms and reduces neuroinflammation in the CNS of P301S MAPT mice via activation of exercise-associated signaling in the CNS.

